# Multi-frequency bioimpedance in human muscle assessment

**DOI:** 10.14814/phy2.12354

**Published:** 2015-04-20

**Authors:** Else Marie Bartels, Emma Rudbæk Sørensen, Adrian Paul Harrison

**Affiliations:** 1 Department of Rheumatology, The Parker Institute, Copenhagen University HospitalBispebjerg, Frederiksberg, Denmark; 2 Department of Veterinary Clinical & Animal Sciences, Faculty of Health & Medical Sciences, Copenhagen UniversityFrederiksberg, Denmark

**Keywords:** Bioimpedance, biomedical technology assessment, skeletal muscle

## Abstract

Bioimpedance analysis (BIA) is a well-known and tested method for body mass and muscular health assessment. Multi-frequency BIA (mfBIA) equipment now makes it possible to assess a particular muscle as a whole, as well as looking at a muscle at the fiber level. The aim of this study was to test the hypothesis that mfBIA can be used to assess the anatomical, physiological, and metabolic state of skeletal muscles. mfBIA measurements focusing on impedance, resistance, reactance, phase angle, center frequency, membrane capacitance, and both extracellular and intracellular resistance were carried out. Eight healthy human control subjects and three selected cases were examined to demonstrate the extent to which this method may be used clinically, and in relation to training in sport. The electrode setup is shown to affect the mfBIA parameters recorded. Our recommendation is the use of noble metal electrodes in connection with a conductance paste to accommodate the typical BIA frequencies, and to facilitate accurate impedance and resistance measurements. The use of mfBIA parameters, often in conjunction with each other, can be used to reveal indications of contralateral muscle loss, extracellular fluid differences, contracted state, and cell transport/metabolic activity, which relate to muscle performance. Our findings indicate that mfBIA provides a noninvasive, easily measurable and very precise momentary assessment of skeletal muscles.

## Introduction

### Background

Bioimpedance has become an accepted and validated technique for use with an array of patients (Salinari et al. 2002; Ivorra 2003; Stahn et al. 2008; Carter et al. 2009; Rebeyrol et al. 2010; Davenport 2013; Kim and Kim 2013; Nescolarde et al. 2013a,b), the method has been tested against MRI (Salinari et al. 2002; Nescolarde et al. 2013a), although its true potential has yet to be reached. With the advent of multi-frequency BIA (mfBIA) units, it is now possible to examine not only the low-frequency end of the Cole–Cole plot (for further interpretation of Cole–Cole plots see Martinsen and Grimnes 2008; section 8.2.8) – the α-dispersion range, where extracellular fluid is the primary focus – but also the β-dispersion range of much higher frequencies (Martinsen and Grimnes 2008; Chapters 3 and 8). The α-dispersion range focuses on the pathway taken by a low-frequency current around the cells *via* the extracellular fluid (Ivorra 2003), and as such provides information regarding dehydration (elevated resistance (*R*)) or edema (lowered *R*). The β-dispersion range focuses on the pathway taken by high-frequency current, which is forced to enter cells, transverse the cell membrane and the intracellular matrix, and continue through adjacent cells. As such the β-dispersion data provide information regarding cell health and cell integrity, more specifically reactance (Xc), membrane capacitance (Mc), and intracellular resistance (Ri) (Ivorra 2003; Grimnes and Martinsen 2008).

### BIA and mfBIA

Bioimpedance analysis (BIA) uses the components of impedance (*Z*): *R*, which is the opposition to the flow of an alternating current through intra-and extracellular ionic solutions, and Xc, which is the delay in the passage of current through the cell membranes and tissue interfaces (Van Der Aa Kuhle et al. 2006). Resistance is inversely related to the fluid content, and Xc indicates cell membrane mass, function and interface. BIA therefore enables characterization or classification of relative changes in hydration and cell health/damage in a noninvasive fashion (Nescolarde et al. 2013a,b).

Newer BIA units (Moon et al. 2008; Lukaski 2013) measure, aside from *R* and *Z*, the centre frequency (fc), a value that defines the kHz needed to obtain a maximum Xc value. fc provides important information regarding the relative density of muscle tissue, for example, more or less contracted at rest. This is due to the fact that a contracted muscle is relatively more dense than a relaxed one, and as such requires a higher frequency to attain the maximal Xc value. In addition, values for membrane capacitance (Mc) are achievable, thus an indication of the cell transport activity of a muscle can also be obtained. The cell membrane is comprised of a lipid bilayer with specific transporter proteins inserted. This structure enables water and large lipids to pass through it. It is otherwise completely closed for other transport. In principle this means that the cell membrane functions like a dielectric. Every cell membrane has both an extracellular and intracellular fluid, thus cell membranes function as a conductor-dielectric-conductor structure, that is to say a capacitor. However, due to the fact that these cell membranes have embedded proteins many of which are ion channels and pumps, the capacitance of the membrane can be affected when transport through any one or multiple channels is permitted.

Furthermore, newer BIA units also allow for a more precise measurement of the extracellular resistance (Re) and the intracellular resistance (Ri), allowing an assessment of localized swelling, dehydration, and muscle damage. Indeed, a relatively recent paper by Stahn et al. (2008) has shown a very high correlation (*r* = 0.89) between Ri and VO_2max_ in a cohort of 115 including 63 men and 52 women. This is an important finding, as it links cellular oxygen consumption at rest with other parameters obtainable when using mfBIA.

### Aims

The aim of this study was to demonstrate the greater potential of the mfBIA technique *via* a number of selected cases, which serve to illustrate the aforementioned parameters in subjects with a known anamnesis. Our hypothesis was that “multi-frequency BIA is capable of detecting contralateral differences in muscle mass, fiber health and level of contraction and metabolic state”. It was also our intention to show how the mfBIA technique is electrode sensitive and present other factors that can influence mfBIA recordings.

Overall, we wished to illustrate how a much more in depth application of the use of mfBIA can be a powerful diagnostic and noninvasive tool in both medical and sports practices.

## Materials and Methods

### Subjects

mfBIA is an accepted measuring technique used routinely in the clinic as well as in the sports field. It is safe and noninvasive. All subjects were informed about this technique, and gave their consent prior to their participation in this study. The study complied with the Helsinki Declaration. The aim of this study has been to provide observational data that highlight the need for a more detailed assessment and presentation of mfBIA measurements in human subjects.

#### Selected subjects

##### Subject #1

The subject was a 65-year-old healthy woman, height 170 cm, weight 58.5 kg. The subject was hyper-mobile, but without any disability connected with this. The subject was fit and well-trained. This subject was used to demonstrate the effects of lying down, sitting and standing, as well as the comparability of Ag/AgCl and platinum electrodes with gel and conductance paste on the BIA recordings.

##### Subject #2

The subject was a 48-year-old man, height 182 cm, weight 95 kg. The subject was chosen due to the fact that he had suffered inflammation of the Achilles tendon in the right leg over a period of 1.5 years, and whilst in recovery after injection with glucocorticoids directly into the tendon, was assessed as having less muscle mass in the injured leg.

##### Subject #3

The subject was a 28-year-old man, height 186 cm, weight 78 kg. The subject was chosen as an example of recent muscle damage since he had suffered a minor muscle injury whilst playing football in his leisure time 2 weeks prior to the measurement. The injured right leg was assessed as having less muscle mass.

#### Healthy young subjects

Eight healthy female handball players, in the youth team (15–17 years), all with a BMI in the normal range, from Ullerslev, Denmark, participated in this study in order to provide a healthy control data set. These girls were not top professional athletes, but were routinely trained twice a week to a high physical standard, with a view to competing at a county level.

### Bioimpedance recordings

**Figure 1:**
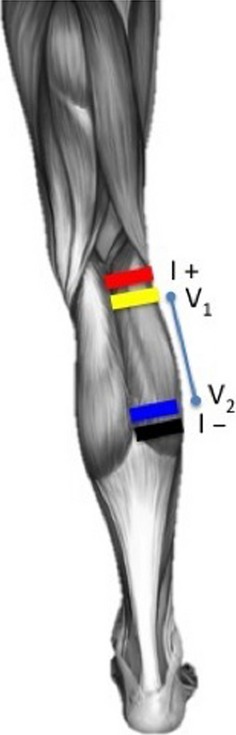
A drawing of the typical electrode setup recommended by the manufacturer and adopted in this study. Note that the current (I+ and I−) electrodes are the outermost electrodes (red and black), whilst the voltage electrodes (V_1_ and V_2_) are innermost (yellow and blue). This drawing is copyright © AH – permission is given for publication.

Platinum electrodes (10 mm × 25 mm) in connection with a conductive paste (Ten20, Weaver & Co, Aurora, CO) was used for BIA-measurements over the *m. Gastrocnemius* region of each leg (see Fig.1). The current electrodes were placed outer-most and the voltage-sensing electrodes 10 mm inside away from them, according to the manufacturers’ recommendations, and the guidelines for electrode placement published in Grimnes and Martinsen (2008), Chapter 6.

The subjects were recorded within a minute of electrode placement, whilst ensuring that they did not make contact with any metal objects. A change in subject position was well within a time frame of 2 min. Measurements were taken using an ImpediMed Inc tetra polar bioimpedance spectroscopy unit (Pinkenba, Qld, Australia). This device, which applies a constant current, scans 256 frequencies between 4 kHz and 1000 kHz, repeating this procedure six times with a 1 sec interval. Using this approach, any slight movement artifacts or changes in the resistance and reactance values due to cable movement, change in the stance, body or electrode movement were minimized.

The subjects were in a standing position with equal weight on both legs, and in the case of Human # 1 & 3, recordings were also made in a sitting position with both legs on the floor. In Human #1, recordings were additionally made in a sitting position without foot contact to the floor, and in a lying (supine) position.

Electrode choice may affect the measurements by creating a capacitance between the electrode surface and the skin. In Human #1, in order to assess electrode-type effects on mfBIA recordings, two types of Ag/AgCl electrodes were also used (Ambu – Blue Sensor, DK-2750, Ballerup, Denmark). The one, N-type with integrated gel, was 44 mm × 22 mm, and the other, an SU-type with gel removed to expose the electrode, was 49 mm × 33 mm. The SU-type electrode surface was attached to the skin with the same conductance paste (Ten20) as used previously with the platinum electrodes. The positions of the Ag/AgCl electrodes were the same as those for the platinum electrodes. Recordings were taken within a 3 min time frame of each other.

### BIA data handling

The BIA recordings were analyzed using the ImpediMed Inc software. The Cole–Cole plot was assessed at the time of recording for a normal distribution, and both the *R* and Xc plots were examined to ensure a precise recording. Subsequent detailed analysis was performed at 50 kHz, where not only the standard parameters of *Z*, *R,* and Xc were measured, but also the Phase Angle (PA) was calculated (arctan Xc/*R*), and the fc, Re, Mc, and Ri parameters were measured.

## Results

### Bioimpedance recordings and analysis

**Figure 2:**
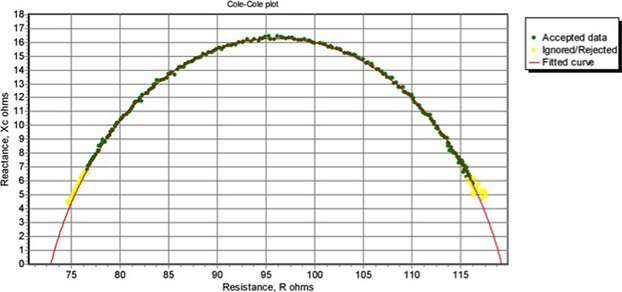
A typical Cole–Cole plot of the mfBIA data obtained for Human #1 in a relaxed state. Note the thick and slightly irregular part of the curve where the measured mfBIA data closely fit to the thinner predicted curve. The fc value is the frequency at which the maximal Xc (peak of curve) is measurable.

An example of data recorded by the mfBIA unit from Human #1 revealed typical signal values (Fig.2). The signals exhibited a clear Cole–Cole plot, a widely used semi-circular graphical representation of frequency-dependent complex dielectric functions such as impedance, reactance, and resistance for the 256 frequencies used. This enables the collection of normal resistance and reactance data for the *m. Gastrocnemius* of the subject. These plots also enabled a detailed analysis of the α-dispersion range (centered around 100 Hz), as well as the β-dispersion range (10 kHz–10 MHz).

#### Effect of electrode type

Table1 shows recordings taken from the *m. Gastrocnemius* of Human #1 with three different electrode types.

**Table 1:** Human #1 – mfBioimpedance values for the left *m. Gastrocnemius* taken with the subject standing free, using platinum electrodes and Ten20 conductance paste, Ag/AgCl ECG electrodes with gel (Medicotest N-type), or Ag/AgCl ECG electrodes (Medicotest SU-type) without gel but with Ten20 conductance paste.

BIA	Platinum + conductance paste	Ag/AgCl + gel	Ag/AgCl + conductance paste
*Z* (Ω)	126.70	114.20	84.10
*R* (Ω)	125.40	112.80	83.00
Xc (Ω)	18.30	17.4	13.60
PA (^o^)	8.30	8.77	9.31
fc (kHz)	54.90	63.4	43.7
Re (Ω)	150.90	130.0	103.90
Mc (pF)	6.88	6.23	13.50
Ri (Ω)	270.80	272.5	165.80

There was a clear effect of electrode type on the *Z*, *R,* and Xc values, with a subsequent effect on PA, which is dependent on both the *R* and Xc values. The *Z* and *R* values were reduced for the N-type Ag/AgCl by approximately 10% *cf* the platinum electrodes, and Xc was affected by approximately 5%. An approximately 29% reduction in the *Z* and *R* values was noted for the SU-type Ag/AgCl + conductance paste *cf* the platinum electrodes, and Xc was affected by approximately 26%. With the SU-type of Ag/AgCl electrodes, the result is an artificially elevated PA compared to that of the platinum electrodes. A similar effect was noted for the Re values, 13% and 31% for the N-type Ag/AgCl and the SU-type Ag/AgCl + conductance paste versus the platinum electrodes, respectively. Mc and Ri were not significantly affected between the N-type Ag/AgCl and platinum electrodes. Generally, the worst effect was found to be with the SU-type Ag/AgCl + conductance paste electrodes. The use of the gel Ag/AgCl electrodes, however, will undoubtedly affect the mfBIA recordings, principally through an effect on the resistivity of the electrode–skin interface (Martinsen and Grimnes 2008, Chapters 2 and 7).

#### Effect of measuring position

The measuring position is especially important in humans if comparisons with other studies, or values at different time points during recovery of muscles over time, are needed.

Table2 shows changes in mfBIA parameters between sitting and lying down (on the front) for *m. Gastrocnemius* of Human #1, and between standing and sitting in Table3 for Human #3, as well as standing for Human #1 (Table4).

**Table 2:** Human #1 – mfBioimpedance values for the left *m. Gastrocnemius* taken with the subject lying down (on chest), sitting with legs free of the floor, and sitting with legs touching the floor, using platinum electrodes.

BIA	Lying down	Sitting – feet free of floor	Sitting – feet touching floor
*Z* (Ω)	98.80	93.50	86.80
*R* (Ω)	97.30	91.90	85.60
Xc (Ω)	17.10	17.00	14.30
PA (^o^)	9.97	10.48	9.48
fc (kHz)	56.00	53.60	60.30
Re (Ω)	119.20	114.60	103.10
Mc (pF)	9.27	10.41	9.63
Ri (Ω)	187.30	170.50	171.00

**Table 3:** Human #3 – mfBioimpedance values for the left and right *m. Gastrocnemius* taken with the subject sitting with both feet on the floor and standing free, using platinum electrodes and Ten20 conductance paste. This subject had suffered a minor muscle injury in the right leg, sustained whilst playing football.

BIA	Sitting – feet on floor		Standing free	
	Left	Right	Left	Right
*Z* (Ω)	89.00	123.20	84.90	116.50
*R* (Ω)	87.10	121.20	83.30	114.70
Xc (Ω)	18.40	22.20	16.70	20.50
PA (^o^)	11.93	10.38	11.34	10.13
fc (kHz)	35.30	41.3	38.70	45.20
Re (Ω)	119.30	155.50	110.90	144.50
Mc (pF)	16.91	9.50	16.00	9.25
Ri (Ω)	147.00	249.70	146.10	235.90

**Table 4:** Human #1 – mfBioimpedance values for the left and right *m. Gastrocnemius* during free standing with equal weight being placed on both legs, using platinum electrodes.

BIA	Left *m. Gastrocnemius*	Right *m. Gastrocnemius*
*Z* (Ω)	126.70	140.40
*R* (Ω)	125.40	138.80
Xc (Ω)	18.30	20.90
PA (^o^)	8.30	8.56
fc (kHz)	54.90	53.60
Re (Ω)	150.90	168.70
Mc (pF)	6.88	6.55
Ri (Ω)	270.80	284.10

In Human #1 (see Table2) there was a slight but significant fall in *Z* and *R* between lying down and sitting with feet in the air, and between sitting with feet in the air and sitting with feet on the floor. However, all *Z* and *R* values for lying down, sitting with feet in the air and sitting with feet on the floor varied greatly (lower by approximately 22–31%) compared to values obtained during standing. Position also had an impact on the Xc and PA values. The Re values followed the same trends, as did the Ri values, whilst the Mc values increased from standing to sitting with feet on the floor – the lowest values being whilst standing, indicating an active state of contraction in *m. Gastrocnemius* in that position. The very slight changes in *R* and Re from a lying down to a sitting position with feet free of the floor and sitting with feet touching the floor are most likely not explained by changes in body fluid (e.g., plasma and blood) with a change in position. This subject had no history of cardiovascular problems and so fluid pooling in the limbs after sitting for such a short period of time seems unlikely.

In Human #3 (see Table3) there was a slight, but consistently lower *Z* and *R* value (approximately 5%) when standing *cf* sitting with feet on the floor. The same was true for the Re value where a 6–7% lower value was seen when standing *cf* sitting. The Ri was also affected in the right leg (injured) *cf* the left – standing versus sitting, a difference of 6%.

The two subjects, Human #1 and #3, showed a different use of their *m. Gastrocnemius* with a change in posture from sitting with their feet on the floor to standing. In both cases a similar delta is noted for *Z* and *R*, although in Human #1 the change is a reduction with sitting or lying *cf* standing, whilst for Human #3 it is the reverse (see Table4). This observation demonstrates the importance of always measuring subjects and muscles in similar positions when comparisons between measurements are needed. Moreover, the difference in the use of *m. Gastrocnemius* between these two human subjects raises another issue, namely that of individual differences in the specific muscles recruited for different types of movement, or as was the case with this study, maintenance of posture.

#### mfBIA used on healthy well-trained handball players

mfBIA was measured prior to training on the medial head of *m. Gastrocnemius* on both legs in a standing position for eight young and healthy female handball players (see Table5). The symmetry between each leg was recorded, as well as the variation in physical fitness between individuals compared with the group mean. Focus was placed mainly on the PA, since this is a ratio which enables comparison between individuals of different body mass and size.

**Table 5:** Handball Players – mfBioimpedance values for the left and right *m. Gastrocnemius* taken with the subjects standing with both feet on the floor, using platinum electrodes and Ten20 conductance paste. These subjects were measured prior to a handball training session – 2 days after their previous workout. Values are the Mean ± SEM of *n* = 8 female handball players.

BIA	Left *m. Gastrocnemius*	Right *m. Gastrocnemius*
*Z* (Ω)	91.1 ± 2.2	95.5 ± 5.4
*R* (Ω)	90.1 ± 2.1	94.5 ± 5.4
Xc (Ω)	13.5 ± 0.9	13.8 ± 0.9
PA (^o^)	8.5 ± 0.4	8.4 ± 0.5
fc (kHz)	54.7 ± 2.5	54.3 ± 2.6
Re (Ω)	108.9 ± 3.4	113.6 ± 6.2
Mc (pF)	9.7 ± 0.4	9.5 ± 1.0
Ri (Ω)	194.2 ± 5.2	213.4 ± 19.1
Delta (*Z*-*R*) Ω	1.0 ± 0.1	1.0 ± 0.1

Values in general were very similar between the right and left leg, and the delta (*Z*-*R*) also showed no signs of unilateral swelling or edema. The Re values are indicative of a reduced extracellular fluid level, and combined with a very high Ri and low Mc, they are suggestive of a posttraining recovery phase.

#### mfBIA case analyses

Human #1: the mfBIA values for *Z* and *R* in this subject were higher than one might normally expect, principally as a result of muscle contraction due to the fact that this subject is standing and using the *m. Gastrocnemius* for support (see Table4). Of course care should be taken when comparing *Z* and *R* absolute values, and for this reason the phase angle (PA) value has been widely adopted – it being the ratio of Xc/*R* and as such it is independent of body mass and size. However, this subject serves to illustrate a very high degree of symmetry for the PA, fc, Mc, and Ri values. It also reveals that the left leg had lower *Z* and *R* values than the right leg, and that the Xc and Re values were also slightly lower. This subject is left limb orientated, and the slightly lower muscle mass (*Z*) with a lower Xc and Re are commensurate with these values, confirming that the left leg is used more than the right, affecting the Xc slightly more in the left *cf* the right leg. In addition to this, a slightly higher blood flow is seen in the left leg (Re). This subject is also noteworthy from the point of view of the fc values, which although very symmetrical in terms of left versus right are somewhat elevated compared to Human #2 and Human #3 (left leg – unaffected). A higher fc in both *m. Gastrocnemius* muscles is in agreement with the fact that this subject is hypermobile and as such could be expected to use muscles for antigravity support of joints rather than connective tissue. A measurement of the circumference of the right and left legs at the level of the *m. Gastrocnemius*, revealed values of 33.0 and 33.2 cm in circumference, respectively, which again confirms the high degree of symmetry seen between the left and right *m. Gastrocnemius*.

Human #2: the mfBIA values for *Z* and *R* in this subject are higher than one might normally expect, principally as a result of muscle contraction due to the fact that this subject is standing and using the *m. Gastrocnemius* for support (see Table6). However, a closer examination shows that the fc for the right leg *cf* the left is lower, indicating a lower level of contraction in the right leg. Likewise, the Mc is slightly higher for the right leg and the Ri slightly lower, whilst the Xc and PA values are both slightly higher than those in the left leg. Combined, the mfBIA values for the right leg show a lower level of contraction (fc), with less transmembrane transport and a lower anabolic state (Ri), whilst the slightly higher Re value may indicate a reduced flow of blood to this muscle during the contracted state associated with free standing. These mfBIA values and conclusions are in accordance with the fact that this subject prefers to use the left leg for weight bearing since the right leg is more painful after a prolonged period of inflammation of the Achilles tendon, despite the fact that the Achilles tendon has been in recovery for the past 6 months. These findings are further supported by measurement of the circumference of the right and left legs at the level of the *m. Gastrocnemius* showing that the right leg was slightly atrophied, being just 42.1 cm in circumference compared to 43.5 cm for the left leg – a 3.2% reduction.

**Table 6:** Human #2 – mfBioimpedance values for the left and right *m. Gastrocnemius* during free standing with equal weight on both legs, using platinum electrodes. This subject had suffered with Achilles tendon inflammation over a 1.5 year period in the right leg.

BIA	Left *m. Gastrocnemius*	Right *m. Gastrocnemius*
*Z* (Ω)	106.80	113.40
*R* (Ω)	105.70	111.90
Xc (Ω)	15.10	18.50
PA (^o^)	8.13	9.39
fc (kHz)	36.00	29.30
Re (Ω)	132.40	149.40
Mc (pF)	11.25	14.35
Ri (Ω)	260.80	229.70

Human #3: the mfBIA values for *Z* and *R* in this subject are higher than one might normally expect, principally as a result of muscle contraction due to the fact that this subject is standing and using the *m. Gastrocnemius* for support (see Table3). However, the between leg values for *Z* and *R* are very different in this subject. The right leg, which is the dominant leg, has a higher *Z* and *R* both during sitting and standing. This is most likely artificially high as a result of the high Re value, which in combination with an especially high Ri in the right leg, and low Mc, indicates postexercise swelling of the muscle fibers. It is also apparent that the right leg is slightly more contracted at rest (sitting with feet on the floor) and during a period of free standing, than the left leg (fc). Taken in combination, these mfBIA differences are in agreement with the fact that the right leg was injured approximately 2 weeks prior to measurement, and that the subject then played football with an injured leg 7 days after the original injury. The differences indicate a higher level of anabolic activity in the right *cf* left leg. This is commensurate with cellular repair. A measurement of the circumference of the right and left legs at the level of the *m. Gastrocnemius* revealed that the right leg was slightly atrophied, being 38.0 cm compared to 39.5 cm for the left leg – a 3.8% reduction.

## Discussion and Conclusions

This study has tested and affirmed the hypothesis that multi-frequency BIA is capable of detecting contralateral differences in muscle mass, fiber health, and level of contraction and metabolic state. It has also shown that the type of electrode selected for such measurements has an effect on the values obtained, as well as revealed that the position and state of contraction of a body region should be a factor worth considering prior to making such an analysis.t

### Effect of choice of setup

#### Electrodes

Aside from the fact that the placement of the electrodes, as in all physiological setups, is important, the choice of electrodes/electrode contact also plays a major role for reliable results (Bogónez-Franco et al. 2009).

Metal/Metal+Salt electrodes, for example Ag/AgCl, are ideal for ECG and EEG recordings when used with an electrode gel applied between the flat disk of the metal electrode and the skin (Tallgren et al. 2005). The combination of the ionic gel and the metal of the electrode creates a local solution of the metal in the gel at the electrode–skin interface. This results in a nonpolarizable electrode where current in a tissue can pass *via* the ionic gel to the electrode and recording cable (McAdams et al. 1995; Tallgren et al. 2005). With such an electrode, the main component of the impedance is resistive between the body electrolytes and the capacitance of the electrode. However, electrode mismatch, that is, when the contact area to the skin for one or more electrodes becomes reduced, either as a result of one electrode lifting from the skin, or in connection with the use of old electrodes where the gel has dried, can affect the impedance recording during BIA analysis (Bogónez-Franco et al. 2009; Buendía et al. 2012).

In the case of ECG and EMG recordings, many commercial electrodes are designed to handle signal frequencies of 10–150 Hz, and the ionic gel concentration is designed to match these needs. However, with mfBIA, frequencies far in excess of 10 or 150 Hz are employed (e.g., 4–1000 kHz), and the ionic gel suitable for ECG or EMG recordings may not be adequate in such cases (Grimnes and Martinsen 2008, Chapter 7).

Another point of importance is the fact that many biological materials exhibit frequency dispersions (Poon and Choy 1981). The electrical impedance of human skin exhibits a characteristic circular arc locus at various frequencies – in fact identical to that described by the Cole–Cole model (Ivorra 2002; Grimnes and Martinsen 2008, Chapter 8). The reason for this can be attributed to changes in the resistance of the ionic paths through the skin with different frequencies – owing to dielectric relaxation processes of the epidermal stratum corneum (Yamamoto and Yamamoto 1976). When recording transdermally, and especially when a variety of frequencies are involved, one should bear in mind that the electrical impedance of the electrode–skin interface is not a constant.

Thus, there are a number of reasons for selecting the ideal electrode and ionic gel for mfBIA recordings, if one is not to impair the recorded values through resistive issues between the body and the electrode itself.

The choice of platinum or other noble metals as an electrode, together with conductive paste, greatly increases the transfer of electrons across the electrode–skin interface. As such this greatly reduces the impedance with this interface and improves the accuracy of the impedance recording. For Ag/AgCl electrodes to work optimally, it is essential that some of the Ag dissolves into the intermediary gel as a counter ion to Cl which also has to be present in ionic form in the gel, thereby ensuring that the activity of Ag and AgCl can be kept constant (electrode of 2^nd^ kind), while the potential will vary with the Cl^−^ concentration (Grønlund 1966). Thus, a combination of a Ag/AgCl electrode with a conductive paste will result in an impedance at the electrode paste barrier, and without a salt solution (gel) to facilitate electron transfer, this electrode configuration will be rather ineffective.

#### Position of subject

Looking at a comparison between measurements, the position and thereby the state of contraction of a muscle or body region becomes a vital factor affecting the outcome of a mfBIA recording. Ideally one should aim for a relaxed state in a muscle group or limb, but on occasions a contracted state such as standing or lifting etc. can also be informative.

In the case of human subjects, one can always ask an individual to adopt a set position or to relax fully. However, when comparing different studies, the position of subjects has to be taken into account. Some measure their subjects in a relaxed supine position (Rebeyrol et al. 2010; Nescolarde et al. 2013a,b), especially in studies assessing body composition (Zhu et al. 2006; Carter et al. 2009; Ling et al. 2011; Davenport 2013), while others use a standing position where a degree of muscle contraction cannot be avoided (Tonkovic et al. 2000; Kim and Kim 2013).

#### Interpretation of a holistic use of multiple mfBIA parameters

##### *Z* and *R*

A considerable number of published bioimpedance studies present partial values for their recordings, choosing either the *Z* or *R* values, but not both. We feel it is important to present as many of the recorded values as possible, if the bioimpedance unit permits this. For example, a combined assessment of the *Z* and *R* values for a muscle can help determine a swelling/edema or a dehydrated state. A low *R* relative to a typical mean *Z* value indicates a region of swelling or inflammation/edema, whilst a relatively high *R* and *Z* recording for a muscle suggests dehydration. Often one can compare with a contralateral limb or muscle group within a subject, but it is still important that values be compared with a normal range for healthy subjects where possible.

In the case of the handball girl data, we noted very high *Z* and *R* values that are indicative of a mild state of dehydration – typical to that found after a period of sustained exercise. It is known that postexercise, cellular activity at the muscle fiber level results in an osmotic gradient that causes the extracellular fluid to enter the muscle cells, giving rise to the well-known phenomena of postexercise muscle swelling. These events combine to give a temporary increase in the *Z* and *R* values, compared with anticipated values at rest. We wish to point out that other measurements performed on sports athletes are often carried out after rest and in a supine position (Nescolarde et al. 2013a,b) – however, our values for the Xc parameter remain identical with those of other studies. These data point out that *R* and *Z* alone are not a good indicator of muscle health and training level – R_i_ and R_e_ values provide a more comprehensive assessment, particularly in combination with the Mc value.

##### *R*, Xc and PA

It is our opinion that the *R*, Xc, and PA values should be considered together since any change in either the *R* or Xc of a muscle will have a consequence for the PA value – the PA being the result of the arctangent of the reactance divided by the resistance. Furthermore, PA is independent of the size of the subject. Thus, a low resistance in a swollen muscle where the Xc is maintained close to the mean will result in a false increase in the PA. This point is made very clear by the handball girl data (see Table5). The changes in *R* and Xc with fluid removal during peritoneal dialysis in human subjects confirm the effects of edema on these mfBIA parameters (Davenport 2013).

##### Xc and Re

Another useful combination of mfBIA values are the Xc and extracellular resistance (Re) values. An elevated Re can be indicative of dehydration, although it may also be due to an impaired perfusion, perhaps as the result of cardiovascular disease (Tonkovic et al. 2000). In the case of the handball girls the Xc values are very comparable to those of other published studies involving sports athletes (Nescolarde et al. 2013a,b) whilst the R_e_ values are rather elevated which is in accordance with the high *R* and X values. These changes are also in accordance with the high R_i_ value and the low Mc value for these subjects. Combined, these changes indicate a level of cellular activity postexercise that has not yet returned to a resting state. These girls had already been active earlier the same day, and in previous days making them borderline over-trained. This point is further illustrated in the case of Human #3 (see Table3) where an injured leg is compared with a healthy leg. In this case there is a 38% percentage higher *Z* and *R* in the healthy leg *cf* the injured leg. Likewise, the R_e_ is 30% higher, the R_i_ is 70% higher and the Mc is 78% lower in the injured leg *cf* the healthy leg. Overall, these data indicate an injured state in the right leg with high cellular activity to restore and repair damaged muscle fibers compared with the healthy leg.

##### Ri, Re, and Mc

The parameters of membrane capacitance (Mc), which illustrates the status of the membrane potential, and hence muscle fiber health as well as the transport status of the membrane, and intracellular resistance (Ri), which has been tightly correlated with VO_2_-max at rest – providing an index of anabolic status of muscles (Stahn et al. 2008), are useful parameters to consider in conjunction with the extracellular resistance.

In Human #3 (see Table3) the Re and Ri values for the right leg are relatively high compared to the left leg, whilst the Mc is relatively low. This is in compliance with the fact that the right leg was injured approximately 2 weeks prior to measurement and subsequently used a week later for football training. The elevated Ri value indicates an anabolic process in the muscle, using oxygen to repair the damaged tissue, and accumulating proteins and other compounds necessary for the repair. The low Mc value is commensurate with a high Ri during cellular repair, as muscle fibers actively transport compounds in and out of the fibers and thereby compromise the membrane potential. In cases of such cellular repair, the accumulation of proteins and other compounds within the recovering cells alters the osmotic gradient in favor of a flow of water into the active cells (Loenneke et al. 2012). Such a change in the osmotic gradient results in a loss of extracellular fluid to the muscle fibers themselves (Sigmund et al. 2014), and as a consequence the Re becomes elevated.

It should be noted, however, that insulin induces an uptake of glucose into muscle cells, thereby altering the osmotic balance and causing cell swelling with a reduction in extracellular volume. This can be seen after a prolonged period of strenuous exercise (Branth et al. 2009).

##### fc

fc is the frequency at which the maximal Xc is recorded on a Cole–Cole plot of resistance against reactance (Ivorra 2003). It is indicative of the amount of energy required to send a constant current through the tissue – the greater the density or level of contraction of the tissue, the higher the energy/frequency needed, and conversely, a less dense tissue, that is, lower level of contraction, the lower the energy/frequency required. Thus, a fall in the fc of a given tissue over a short period of time is indicative of a less dense tissue, and in the case of muscle, this indicates a more relaxed state. In Human #2 there is a clear difference in the fc value with the injured right leg showing a higher fc than the healthy left leg (15% difference), the leg that was bearing more of the body′s weight. It is also noteworthy that for Human #1, a healthy but hypermobile subject, the fc is elevated compared with Human #3 (42% difference in the sitting position), but almost identical, when standing, to values obtained for the physically active handball girls. This is indicative of a higher level of muscle contraction whilst standing, most likely as a result of less support from the connective tissue associated with joint and balance stabilization in this subject.

## Conclusion

We conclude that mfBIA is a useable noninvasive technique enabling a “snap-shot” assessment of skeletal muscle in terms of anatomical, physiological, and pathological state. This implies the use of all the possible parameters attainable with an mfBIA unit. The technique is easily applicable in the clinic or sports setting, but one must consider electrode type as well as the position of the subject when wishing to compare data with other studies or between clinics.
